# Molecular Recognition of Human Liver Cancer Cells Using DNA Aptamers Generated via Cell-SELEX

**DOI:** 10.1371/journal.pone.0125863

**Published:** 2015-05-04

**Authors:** Jiehua Xu, I-Ting Teng, Liqin Zhang, Stefanie Delgado, Carole Champanhac, Sena Cansiz, Cuichen Wu, Hong Shan, Weihong Tan

**Affiliations:** 1 Department of Nuclear Medicine, The Third Affiliated Hospital, Sun Yat-Sen University, Guangzhou, Guangdong, China; 2 Department of Chemistry, Department of Biochemistry and Molecular Biology and Department of Physiology and Functional Genomics, Center for Research at the Bio/Nano Interface, Health Cancer Center, UF Genetics Institute and McKnight Brain Institute, University of Florida, Gainesville, FL, United States of America; 3 Interventional Radiology Institute, Department of Radiology, The Third Affiliated Hospital, Sun Yat-Sen University, Guangzhou, Guangdong, China; Consiglio Nazionale delle Ricerche (CNR), ITALY

## Abstract

Most clinical cases of liver cancer cannot be diagnosed until they have evolved to an advanced stage, thus resulting in high mortality. It is well recognized that the implementation of early detection methods and the development of targeted therapies for liver cancer are essential to reducing the high mortality rates associated with this disease. To achieve these goals, molecular probes capable of recognizing liver cancer cell-specific targets are needed. Here we describe a panel of aptamers able to distinguish hepatocarcinoma from normal liver cells. The aptamers, which were selected by cell-based SELEX (Systematic Evolution of Ligands by Exponential Enrichment), have Kd values in the range of 64-349 nM toward the target human hepatoma cell HepG2, and also recognize ovarian cancer cells and lung adenocarcinoma. The proteinase treatment experiment indicated that all aptamers could recognize target HepG2 cells through surface proteins. This outcome suggested that these aptamers could be used as potential probes for further research in cancer studies, such as developing early detection assays, targeted therapies, and imaging agents, as well as for the investigation of common membrane proteins in these distinguishable cancers.

## Introduction

Liver cancer is the sixth most common cancer in the world and the third leading cause of cancer-related death [1], resulting in 0.7 million deaths annually. As the largest internal organ and the largest gland in the human body, the liver serves many vital functions, including breaking down and storing nutrients required for energy production or tissue repair, filtering and degrading toxic wastes in the blood, synthesizing most of the clotting factors that keep the body from massive bleeding, and producing chemicals and hormones necessary for regulating many bodily functions. Despite this critical role, the development of liver cancer is rarely diagnosed in its early stages because, in most cases, the signs and symptoms do not appear until the later stages, making it a highly lethal malignancy with a small 5-year survival rate. Thus, developing early detection methods and advanced targeted therapies is essential in fighting liver cancer.

Aptamers are short, single-stranded DNA or RNA oligonucleotides capable of specific binding to a range of corresponding target molecules with high affinity. The method of generating aptamers called SELEX (Systematic Evolution of Ligands by Exponential Enrichment) [2, 3] follows a series of steps: 1) chemical synthesis of an oligonucleotide library having 10^13^–10^16^ single-stranded nucleic acid molecules, 2) direct exposure of the library to the targets to differentiate binding strands from spectators, 3) extraction and amplification of survivors, 4) enrichment of the aptamer survivors by iterative rounds, and, finally, 5) sequencing to identify individual candidates.

The SELEX technology was further developed in our lab to utilize whole cells as targets in the aptamer selection process. The cell-SELEX process ensures that candidate oligonucleotides bind to the native state of the protein targets on the cancer cell surface [4]. Using cell-SELEX, aptamers can be generated for diseased cells without prior knowledge of a given target’s molecular signature, thus making it possible to discover molecular probes for diseases with heretofore unknown biomarkers, which can subsequently be identified using chemical and molecular biological methods [5, 6]. A number of aptamers capable of recognizing different cell types, including red blood cells (RBCs) [7], and cells for lymphocytic leukemia [4], myeloid leukemia [8], colorectal cancer [9], breast cancer [10], ovarian cancer [11], small cell lung cancer [12], non-small cell lung cancer [13, 14], and pancreatic cancer [15], have all been generated using this method. In addition, several cell surface biomarker-aptamer pairs have been identified, including alkaline phosphatase placental-like 2 (ALPPL-2) [15], Prominin-1 (CD133) [16], epidermal growth factor receptor (EGFR) [17], human epidermal growth factor receptor 2 (HER2) [18, 19], immunoglobin heavy mu chain (IGHM) [20], protein tyrosine kinase 7 (PTK7) [4, 5], and their corresponding aptamers. The discovery of cancer specific aptamers provides great potential in biomedical research and in the development of cell-specific diagnosis and therapeutics [21], especially when cell surface biomarkers are often related to cell regulations or signaling pathways.

As oligonucleotides, aptamers are readily reproducible by chemical synthesis with minimum batch-to-batch variations. Moreover, with chemical modification, it is easy to introduce functional modules onto aptamers to fulfill specific needs, such as fluorophores [22–25], chemical linkers [26, 27], therapeutics [28–30], or even nanoparticles [31–33]. With the above-mentioned remarkable properties, the development of aptamers has been exploited in various fields, particularly those involving biomedical applications [34–41]. Further research has led to enhancement of the specificity and efficacy of delivering imaging, diagnostic, or therapeutic agents with escort aptamers [24, 33, 42].

The aim of the current study was to discover aptamers that recognize hepatocellular carcinoma (HCC), which is the major form of liver cancer, accounting for 90% of all liver cancers [43]. Here, the cell-SELEX method was used to generate aptamers capable of differentiating human hepatocellular liver carcinoma cell line HepG2 from normal liver epithelial cell line THLE-2. These aptamers can be used as tools in further biomedical studies and clinical applications.

## Materials and Methods

### Cell Culture and Buffers

HepG2 (hepatocellular carcinoma) and THLE-2 (normal liver) cells were chosen as positive and negative selection cells, respectively. Other cell lines, including HeLa (cervical cancer), MCF-7 and MDA-MB-231 (breast adenocarcinoma), PL45 (pancreatic cancer), H226 (lung squamous carcinoma), A549 (lung adenocarcinoma), Ramos (Burkitt’s lymphoma), CCRF-CEM (T cell leukemia), and TOV-21G (ovarian cancer), were used for examining the selectivity of the aptamer candidates. All cell lines were purchased from the American Tissue Culture Collection (ATCC). HepG2, A549, MCF-7, and PL45 were maintained in Dulbecco’s modified Eagle’s medium (DMEM); Ramos, CCRF-CEM, H226 and HeLa cells were maintained in RPMI-1640. The TOV-21G cell line was maintained in culture with MCBD 105: Medium 199 (1:1). All above media were purchased from Sigma-Aldrich. The MDA-MB-231 cell line was cultured in Leibovitz's L-15 Medium (ATCC) at 37°C and prevented from contacting CO_2_. BEGM Bullet Kit (Lonza/Clonetics Corporation) was used to prepare growth medium for THLE-2 cells. Gentamycin/Amphotericin (GA) and Epinephrine were discarded from the kit but an extra 5 ng/mL of epidermal growth factor (EGF), 70 ng/mL of phosphoethanolamine, and all other additives in the kit were included in basal medium. All media were supplemented with 10% heat-inactivated fetal bovine serum (FBS) (Gibco) and 1% penicillin-streptomycin (100 units/mL of penicillin and 100 mg/mL of streptomycin) (Gibco). All cells, except for MDA-MB-231, were incubated at 37°C under a 5% CO_2_ atmosphere.

Washing buffer was prepared from Dulbecco’s phosphate buffered saline (PBS) with calcium chloride and magnesium chloride (Sigma-Aldrich) with additional glucose (4.5 g/L) and MgCl_2_ (5 mM). Binding buffer was prepared by adding yeast tRNA (0.1 mg/mL) (Sigma-Aldrich) and BSA (1 mg/mL) (Fisher) to the washing buffer.

### Synthesis and Purification of DNA Library and Primers

The forward and the reverse primers were labeled with FAM and biotin, respectively, at their 5'-ends. The sequence of the forward primer was 5’-FAM-AGA GAC CCT GAC TGC GAA-3’; the sequence of the reverse primer was 5’-Biotin-AAG AAG CCA CCG TGT CCA-3’. The DNA library consisted of a randomized 25-nt region flanked by primer binding sites: 5’-AGA GAC CCT GAC TGC GAA-(N_25_)-TGG ACA CGG TGG CTT CTT-3’. All library and primer sequences were purchased from Integrated DNA Technologies (IDT) and purified by reverse phase HPLC.

### Polymerase Chain Reaction

PCR amplification parameters were optimized before the selection process. All PCR mixtures contained 50 mM KCl, 10 mM Tris-HCl (pH 8.3), 1.5 mM MgCl_2_, dNTPs (0.2 mM each), 0.5 μM each primer, and Hot start Taq DNA polymerase (0.015 units/μL). PCR was performed on either a BioRad T100 or C1000 Thermo Cycler, and all PCR reagents were purchased from Takara. Each amplification cycle was performed at 90°C for 30 sec, 57°C for 30 sec, and 72°C for 30 sec, followed by a final extension for 3 min at 72°C.

### *In Vitro* Selection

The cell-SELEX process was performed based on the protocol [44] developed by our group with some modifications. The selection was performed on cell monolayers, as both positive HepG2 and negative THLE-2 used here are adherent cell lines. For the first round of the selection, the DNA pool consisted of 20 nmol of the oligonucleotide library in 700 mL of binding buffer. For the later rounds, 250 nM of oligonucleotides amplified from the previous remaining pool were used. The DNA library was heated at 95°C for five minutes, followed by rapid cooling on ice for 5 minutes before incubation, allowing the DNA sequences to form the most favorable secondary structures. The DNA library was then incubated with monolayer HepG2 cells for 1 hour at 4°C after removal of medium and washing twice with washing buffer. The incubation time decreased as the selection progressed: 60 min for rounds 1 and 2, 45 min for round 3, and 30 min for all subsequent rounds. Between each cycle, the cells were washed three times with washing buffer to remove unbound sequences. The washing time and the volume of washing buffer were increased as the selection progressed to remove weak candidates and obtain aptamers with high specificity and selectivity. The cells were then detached from the dish with a cell scraper, and the debris was collected in 500 μL of binding buffer. The complex was heated at 95°C for 10 minutes and then centrifuged at 14,000 rpm for 5 minutes. The supernatant was collected and was ready for PCR amplification during the first two rounds when no negative selection was included. For later rounds with negative selection, the supernatant was incubated with the monolayer of negative cells THLE-2 for 1 hour at 4°C, and the supernatants were collected.

The supernatant containing bound DNA sequences was amplified by PCR using FAM- and biotin-labeled primers. The number of optimized PCR amplification cycles was confirmed with agarose gel electrophoresis. Streptavidin-coated sepharose beads (GE Healthcare Life Sciences) were used to isolate the PCR products from the reaction mixture. The fluorophore-labeled single-stranded DNA (ssDNA) was then separated from the biotinylated antisense ssDNA by eluting with 200 mM NaOH. Finally, the ssDNA was desalted with a NAP-5 column (GE) and redissolved in binding buffer.

The entire selection process was repeated until a sustained significant enrichment was obtained at the 19th round. The enrichment of the pools was analyzed with flow cytometry (Accuri C6, BD).

### Next-generation Sequencing and Analysis

Enriched pool 19 was chosen for sequencing. The ssDNA separated from pool 19 was PCR-amplified again to add the adapter sequences (CCA TCT CAT CCC TGC GTG TCT CCG TCT CCG ACT CAG AGA GAC CCT GAC TGC GAA and CCT CTC TAT GGG CAG TCG GTG ATA AGA AGC CAC CGT GTC CA) and purified using a PCR purification kit (Qiagen). The purified samples were submitted to Ion Torrent next-generation sequencing at the University of Florida, ICBR Sequencing Core Facility. The products were aligned to identify the most abundant sequences using Galaxy software. Any with fewer than 10 copies were removed from the analysis. The remaining reads (501,084) were then clustered, and the 20 most abundant sequences (Table S1 in S1 File) were chemically synthesized for further characterization.

### Binding Analysis

Recovered aptamer candidates were synthesized by the standard phosphoramidite method using a 3400 DNA synthesizer (Applied Biosystems) and purified by reversed phase HPLC (Varian Prostar using a C18 column and acetonitrile/triethylammonium acetate as the mobile phase). Reagents for synthesis were purchased from Glen Research. BD Accuri C6 flow cytometry (BD Immunocytometry Systems) was applied to monitor the enrichment of ssDNA sequences in the pools during the selection process and to evaluate the binding affinity and specificity of the selected aptamer candidates. Non-enzymatic cell dissociation solution was used to detach cells, and the dispersed cells were then washed with washing buffer. When protease-treated cells were needed, trypsin was used instead of non-enzymatic cell dissociation solution. Trypsin and non-enzymatic cell dissociation solution were both purchased from Sigma-Aldrich. Binding assays were performed by flow cytometry after incubating 4 x10^5^ dispersed cells in 200 μL binding buffer with pools or aptamers at 250 nM for 30 min at 4°C (or 37°C as indicated). For each aptamer candidate, the binding affinity (as K_d_) towards target cell line HepG2 was evaluated using Sigma Plot by fitting the relative mean fluorescence intensity of binding versus the concentration of the aptamers using the saturation equation Y = B_max_X/(K_d_ +X) (**Y** is specific binding, at the concentration of aptamer = **X** in nanomolar, and Bmax is maximal binding.) Cells were incubated at 4°C for 30 min with a series of concentrations (0.1, 0.5, 2, 5, 10, 20, 50, 100, 250, 500, 1000, 2000 nM) of each recovered aptamer with biotin labeled at the 5'-end. Cells were then washed twice with 1 mL of washing buffer, suspended in 200 μL of binding buffer containing streptavidin-PE, and stained for 20 min. Cells were again washed twice with 1 mL of washing buffer and then suspended in 100 μL of binding buffer for flow cytometric analysis. The biotin-labeled unselected library was used as a negative control to determine the background binding. The mean fluorescence intensity of the unselected library was subtracted from that of the corresponding aptamer with the target cells to determine the specific binding of the labeled aptamer. All binding assays were repeated three times.

## Results and Discussion

**Fig 1:**
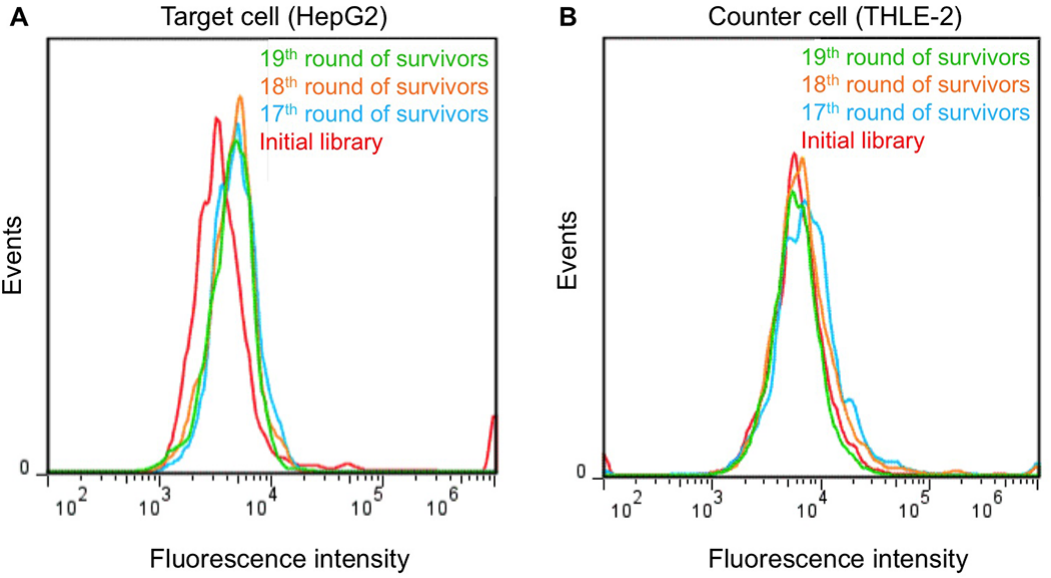
Monitoring the progress of cell-SELEX using flow cytometry. (A) Binding assays showed a noticeable shift in bulk binding of the pool to liver cancer cells (HepG2) and stopped increasing after the 17^th^ round of selection. (B) A subtle decrease in fluorescence intensity was observed for the normal liver cells (THLE-2), reaching a minimum shift in the 19^th^ round. The red curve represents cells incubated with unselected initial library.

The selection process began with a random library containing approximately 1.2×10^16^ (20 nmol) ssDNA sequences of 61 nucleotides (nt), followed by sequential binding with the target HepG2 cells, elution and subsequent PCR amplification for a total of 19 rounds. Counter-selection with THLE-2 cells was introduced in the third round and carried out in all following rounds to eliminate the possibility of any oligonucleotides recognizing common surface markers on both target and negative cell lines. Enrichment progress was monitored by testing the binding of recovered ssDNA from each round on target cells using flow cytometry. Gradual shifts in fluorescence intensity were observed in subsequent rounds. The fluorescent signal stopped increasing between rounds 17 and 19, implying that saturated binding of the enriched pools had been achieved (Fig 1A). No obvious fluorescence intensity increase was found with normal THLE-2 liver cells in pool 19 (Fig 1B), indicating that, compared to the initial library, enriched ssDNA pool 19 showed preferential binding to HepG2 cells, not THLE-2 cells.

The ssDNA recovered from round 19 was submitted for next-generation deep sequencing. The most abundant 20 sequences (Table S1 in S1 File) were chemically synthesized, labeled with biotin at the 5’ end, and then purified by HPLC. The sequences were quantified (UV 260/280) and diluted to standard concentrations.

**Fig 2:**
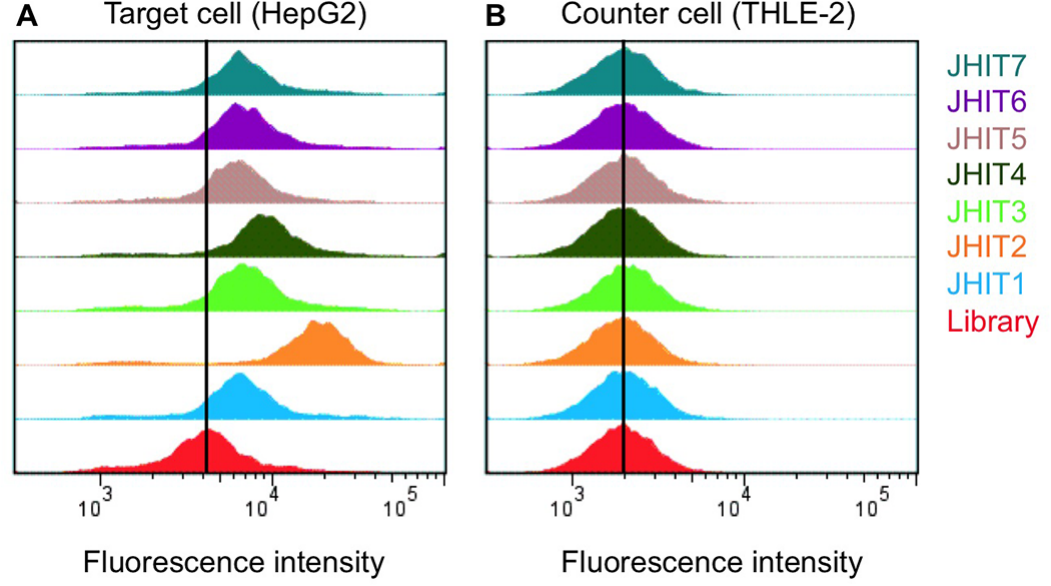
Binding and specificity of selected DNA aptamers. The selected aptamers showed apparent binding to hepatoma HepG2 cells (A), while no fluorescence enhancement was found with normal epithelial liver cells (B), using flow cytometry. Experiments were performed at 4°C.

Binding analysis was performed by incubating positive or negative cells with 250 nM of each aptamer candidate. According to flow cytometric analysis results, none of the oligonucleotides displayed binding with THLE-2 normal epithelial liver cells, while the seven aptamer candidates showed apparent shifts in fluorescence intensity on HepG2 cells with respect to a random sequence (Fig 2, Table S2 in S1 File), implying that all the selected aptamers could differentiate liver cancer cells from normal liver cells.

The binding affinities of the selected aptamers were then determined by evaluating the dissociation constants (K_d_). A smaller K_d_ value indicates stronger binding of the selected aptamer to the target cells. As listed in Table 1, the aptamers reported in this study showed binding affinities toward HepG2 cells in the nanomolar range (64–349 nM), suggesting that these aptamers bound tightly to their target HepG2 cells.

**Table 1:** Aptamer sequences (showing only the randomized region) and their dissociation constants (K_d_).

Name	Sequences	K_d_ (nM)
JHIT1	5’ CCC AAA TCG CAC TCC ATC CCC TAC A 3’	207±35
JHIT2	5’ CCC AAT CGC ACC ACA TCT CAA CAT G 3’	64±11
JHIT3	5’ CTC CAA CTG AGC TCC ATC CCC TAC A 3’	272±23
JHIT4	5’ CCC ACT TCG CAC CAC TCC TCT ACA G 3’	349±70
JHIT5	5’ CCC AAT TCG CGT TCC ATC CCC TAC A 3’	334±48
JHIT6	5’ CTC AAC TCG CAA TGT CCA CCT CTA C 3’	249±84
JHIT7	5’ CCC ATA TCG CAT TTC CAT CCC AAC A 3’	289±56

**Fig 3:**
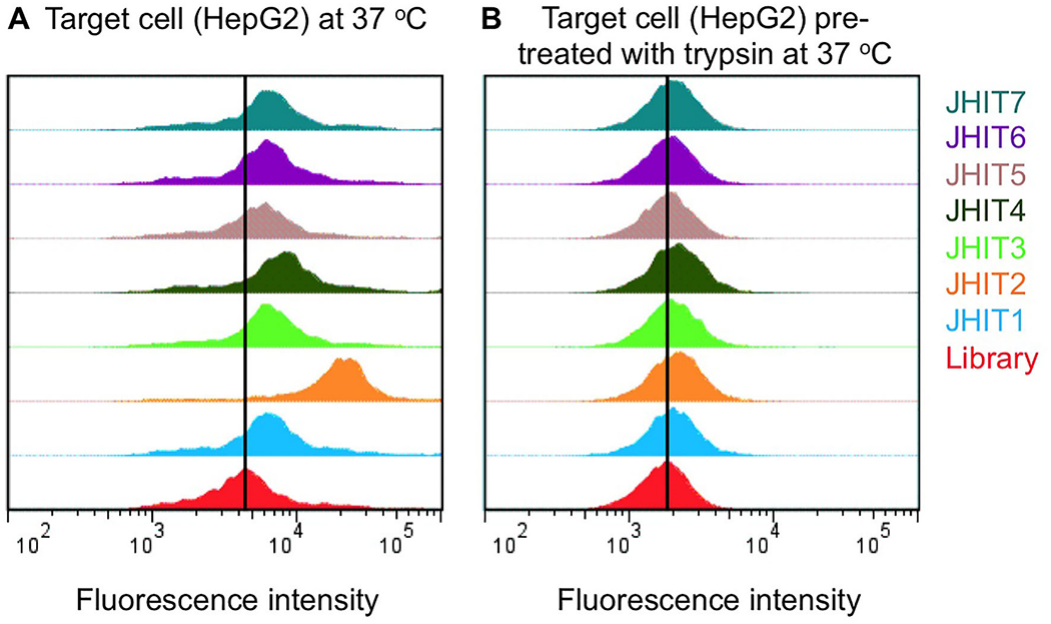
Binding of selected aptamers at physiological temperature without and with trypsin pretreatment. (A) The shift of fluorescence intensity on HepG2 incubated with each of the selected aptamers maintained at 37°C. (B) The fluorescence intensities did not shift when the HepG2 cells were treated with trypsin for 30 min prior to incubation, indicating that membrane proteins are the likely targets.

To prevent the binding sequences from being internalized, we performed the *in vitro* selection at 4°C, as well as the binding assays mentioned above. The selected aptamers did not lose their recognition to target HepG2 cells at physiological temperature (Fig 3A). The fluorescence shift from cells incubated with aptamers compared to cells incubated with the library was conserved when the binding tests were carried out at 37°C.

It has been reported that aptamers specifically interact with the surfaces of target cells during selection [5, 20]; therefore, another set of binding tests (37°C) using HepG2 cells treated with trypsin for 30 min prior to incubation with probes was performed to examine if the selected aptamers were targeting membrane proteins on HepG2. As shown in Fig 3B, all selected aptamers lost their preferential binding to the library with protease-treated HepG2 cells, indicating the targets of these aptamers are likely to be membrane proteins.

It has been reported that certain proteins are cancer-associated [45, 46]. Therefore, identifying the common proteins presented by cancer cells and investigating their relevance to cancers may provide clues about cancer development. After proving the selected aptamers were capable of differentiating hepatocellular carcinoma from normal liver epithelial cells, we further examined the binding selectivity of the aptamers against cell lines for other types of cancer, including lung squamous carcinoma (H226), lung adenocarcinoma (A549), cervical adenocarcinoma (HeLa), breast adenocarcinoma (MCF-7, MDA-MB-231), ovarian adenocarcinoma (TOV-21G), pancreatic adenocarcinoma (PL45), and leukemia (Ramos, CCRF-CEM) (Table 2). It was found that the seven aptamers also displayed significant binding towards lung adenocarcinoma, ovarian cancer and embryonic kidney cells, indicating the strong possibility of some common proteins expressed by these cells, while, at the same time, they showed no affinity to leukemia or cervical cancer cells. Since lung cancer is a common destination of liver cancer metastasis [47], this result could support the use of these aptamers as molecular probes to study cancer metastasis. In addition, all the aptamers exhibited recognition towards one breast cancer cell line, MCF-7, but not the other breast cancer cell line, MDA-MB-231. This difference can be attributed to the fact that they are two distinct subtypes in breast cancer. For instance, MCF-7 falls in the Luminal A subtype with highly expressed estrogen receptor (ER) and progesterone receptor (PR), and MDA-MB-231 belongs to the Basal-like subtype which is negative for ER and PR [48]. Accordingly, this result suggests that these aptamers may be applied to hormone-dependent cancer studies.

**Table 2:** Binding of selected aptamers to different cell lines.

Cell line*	JHIT1	JHIT2	JHIT3	JHIT4	JHIT5	JHIT6	JHIT7
HepG2	+++	+	++	+	++	+	+
THLE-2	-	-	-	-	-	-	-
H226	+	+	+	+	+	+	+
A549	++	+++	+++	+	++	++	++
HeLa	-	-	+	-	-	-	-
MCF-7	+	++	+	+	+	+	+
MDA-MB-231	-	-	-	-	-	-	-
TOV-21G	++	+++	+++	++	++	+++	++
PL45	+	+	+	+	-	-	-
Ramos	-	-	-	-	-	-	-
CCRF-CEM	-	-	-	-	-	-	-

A threshold based on the fluorescence intensity of FAM in the flow cytometric analysis was chosen so that 95% of cells incubated with the FAM-labeled unselected DNA library would have lower fluorescence intensity than the threshold. When the FAM-labeled aptamers were allowed to interact with the cells, the percentage of cells with fluorescence above the set threshold was used to evaluate the binding capacity of the aptamer to the cells. 0–10%,-; 11–30%, +; 31–50%, ++; 51–70%, +++; 71–85%, ++++; >86, +++++

*Cell lines: Liver cancer (HepG2), normal liver epithelial cell (THLE-2), lung cancer (squamous carcinoma H226 and adenocarcinoma A549), cervical cancer (HeLa), breast cancer (MCF-7, MDA-MB-231), ovarian cancer (TOV-21G), pancreatic cancer (PL45), leukemia (Ramos, CCRF-CEM).

## Conclusion

All seven aptamers reported here are capable of distinguishing hepatoma from normal liver cells; they demonstrated high affinities towards the HepG2 cell line at 4°C (K_d_’s of 64–349 nM), as well as at 37°C, while no detectable binding for normal liver cells was observed. The proteinase treatment experiments indicated that all seven aptamers recognize target cell HepG2 through surface proteins. In addition, these aptamers showed recognition towards lung cancer, ovarian cancer, and Luminal A subtype breast cancer. These outcomes suggest that these aptamers may be potential probes for further research in cancer studies, such as developing early detection assays, targeted therapies, and imaging agents, as well as for the investigation of common membrane proteins in these distinguishable cancers.

## Supporting Information

S1 File Combined file of supporting tables. **Table S1:** Number of reads and percentage for the 20 most abundant sequences. Sequence # is designated in the order of the abundance. The total # of reads in the entire DNA pool is 501,084. Among the most abundant twenty sequences, seven of them are reported as aptamers targeting HepG2 cells. **Table S2:** Fluorescence intensity detected from cells incubated with 250 nM of each of the aptamers or a random 61-mer DNA library. (G-mean: geometric mean)
(DOCX)
