# Supplementary material for: Molecular Recognition of Human Liver Cancer Cells Using DNA Aptamers Generated via Cell-SELEX
Source: PLoS One. 2015 May 4;10(5):e0125863. doi: 10.1371/journal.pone.0125863 (PMC4418664; doi:10.1371/journal.pone.0125863)
Supplement: S1 File — Table S1: Number of reads and percentage for the 20 most abundant sequences. Sequence # is designated in the order of the abundance. The total # of reads in the entire DNA pool is 501,084. Among the most abundant twenty sequences, seven of them are reported as aptamers targeting HepG2 cells. Table S2: Fluorescence intensity detected from cells incubated with 250 nM of each of the aptamers or a random 61-mer DNA library. (G-mean: geometric mean) (DOCX) [file pone.0125863.s001.docx]

Supplementary Information

| # | Sequences in the randomized region | # of reads | Percentage | Name as aptamer |
| --- | --- | --- | --- | --- |
| 1 | CCGGAAGCACAGTATGTCATCGCAT | 115,151 | 22.98% |  |
| 2 | CCCAAATCGCACTCCATCCCCTACA | 61,312 | 12.24% | JHIT1 |
| 3 | CATCAACTGAGCTCCATCCCCTATA | 56,557 | 11.29% |  |
| 4 | CCCAATCGCACCACATCTCAACATG | 48,543 | 9.69% | JHIT2 |
| 5 | CCGGAAGCACTCTCTTAGTCGCATA | 28,439 | 5.68% |  |
| 6 | CCTAAAGCAGCTCCATTCTCTACTA | 15,556 | 3.10% |  |
| 7 | CTCCAACTGAGCTCCATCCCCTACA | 10,450 | 2.09% | JHIT3 |
| 8 | CCCACTTCGCACCACTCCTCTACAG | 7,376 | 1.47% | JHIT4 |
| 9 | CCCAATTCGCGTTCCATCCCCTACA | 6,644 | 1.33% | JHIT5 |
| 10 | CTCAACTCGCAATGTCCACCTCTAC | 6,005 | 1.20% | JHIT6 |
| 11 | CCCAACTAGCAGCCAATCCCTACAT | 4,912 | 0.98% |  |
| 12 | CCCAATCGCACCATATCTCTACATA | 3,777 | 0.75% |  |
| 13 | CCCAAATCGCACTCCATCCCTACA | 3,564 | 0.71% |  |
| 14 | CCGGAAGCAAAATCCTCAGTCGAAT | 3,561 | 0.71% |  |
| 15 | CCCATATCGCATTTCCATCCCAACA | 2,988 | 0.60% | JHIT7 |
| 16 | CCCAATTCGCACCAAACCTCTACTT | 2,185 | 0.44% |  |
| 17 | CCCATATCGCCTTCCAACCTCTACT | 2,157 | 0.43% |  |
| 18 | CCCTACTCGCAATCCATCCCCTATA | 1,826 | 0.36% |  |
| 19 | TCCCAAATTTGCTCCATCCCCTACA | 1,741 | 0.35% |  |
| 20 | CCCAAATCGCTAGTCCATCCCTACA | 1,680 | 0.34% |  |

**Table S1. Number of reads and percentage for the 20 most abundant sequences.** Sequence # is designated in the order of the abundance. The total # of reads in the entire DNA pool is 501,084. Among the most abundant twenty sequences, seven of them are reported as aptamers targeting HepG2 cells.

| Cell line | Aptamer | Median fluorescence | G-mean fluorescence |
| --- | --- | --- | --- |
| HepG2 | Library | 4.24×10^3^ | 4.33×10^3^ |
|  | JHIT1 | 6.47×10^3^ | 6.29×10^3^ |
|  | JHIT2 | 1.74×10^4^ | 1.37×10^4^ |
|  | JHIT3 | 6.84×10^3^ | 6.65×10^3^ |
|  | JHIT4 | 8.87×10^3^ | 8.19×10^3^ |
|  | JHIT5 | 6.21×10^3^ | 6.04×10^3^ |
|  | JHIT6 | 6.65×10^3^ | 6.71×10^3^ |
|  | JHIT7 | 6.58×10^3^ | 6.59×10^3^ |
| THLE-2 | Library | 1.97×10^3^ | 1.96×10^3^ |
|  | JHIT1 | 2.00×10^3^ | 1.99×10^3^ |
|  | JHIT2 | 1.92×10^3^ | 1.91×10^3^ |
|  | JHIT3 | 2.08×10^3^ | 2.07×10^3^ |
|  | JHIT4 | 1.96×10^3^ | 1.95×10^3^ |
|  | JHIT5 | 1.98×10^3^ | 1.97×10^3^ |
|  | JHIT6 | 1.98×10^3^ | 1.97×10^3^ |
|  | JHIT7 | 2.00×10^3^ | 1.98×10^3^ |

**Table S2. Fluorescence intensity detected from cells incubated with 250 nM of each of the aptamers or a random 61-mer DNA library.** (G-mean: geometric mean)
